# The Practice of Medicine; or a Treatise on Special Pathology and Therapeutics

**Published:** 1842-07

**Authors:** 


Art. IX.—*The Practice of Medicine; or a Treatise on Special Pathology and Therapeutics.* By Robley Dunglison, m.d., Professor of the Institutes of Medicine in Jefferson College.—*Philadelphia,* 1842. Two Vols. 8vo, pp. 572, 750.

The singular success that has attended Dr. Dunglison's various elementary works might, of itself, be sufficient excuse for his publishing in America a new treatise on practical medicine. He has, however, a still better reason in the fact so well stated in his preface, that "the improvements and modifications incessantly taking place in the departments of pathology and therapeutics, render it advisable, from time to time, to incorporate them, so as to furnish those, to whom the different general treatises, monographs, and journals are not accessible, with the means of appreciating their existing condition."

In the volumes before us Dr. Dunglison has proved that his acquaintance with the present facts and doctrines, wheresoever originating, is most extensive and intimate; and the judgment, skill, and impartiality with which the materials of the work have been collated, weighed, arranged, and exposed, are strikingly manifested in every chapter. The author may truly say that "he is not conscious of possessing any exclusive opinions; and has endeavoured to be essentially eclectic; neither is he aware of having any undue prejudices." The consequence of this is that the different parts of the work bear a more equable proportion to one another than is apt to be the case with treatises compiled by writers of great originality, real or imagined. Great care is everywhere taken to indicate the sources of information; and under the head of treatment formulæ of the most appropriate remedies are invariably introduced. In conclusion, we congratulate the students and junior practitioners of America, on possessing in the present volumes a work of standard merit to which they may confidently recur in their doubts and difficulties.

